# Bispecific soluble cytokine receptor-nanobody fusions inhibit Interleukin (IL-)6 trans-signaling and IL-12/23 or tumor necrosis factor (TNF) signaling

**DOI:** 10.1016/j.jbc.2023.105343

**Published:** 2023-10-13

**Authors:** Annika Gesiorowski, Julia Ettich, Julia Werner, Christoph Wittich, Stephan Pieper, Giacomo Padrini, Kristina Behnke, Doreen M. Floss, Philipp A. Lang, Jens M. Moll, Jürgen Scheller

**Affiliations:** 1 Institute of Biochemistry and Molecular Biology II, Medical Faculty, Heinrich-Heine-University, Düsseldorf, Germany; 2 Institute of Molecular Medicine II, Medical Faculty, Heinrich-Heine-University, Düsseldorf, Germany; 3 PROvendis GmbH, Muelheim an der Ruhr, Germany

**Keywords:** Interleukin 6, Interleukin 12, Interleukin 23, tumor necrosis factor, gp130, IL-6 trans-signaling, nanobody, bispecific inhibitor

## Abstract

At least 0.5% of people in the Western world develop inflammatory bowel disease (IBD). While antibodies that block tumor necrosis factor (TNF) α and Interleukin (IL-)23 have been approved for the treatment of IBD, IL-6 antibodies failed in the phase II clinical trial due to non-tolerable side effects. However, two clinical phase II studies suggest that inhibiting IL-6/soluble IL-6R (sIL-6R)-induced trans-signaling *via* the cytokine receptor gp130 benefit IBD patients with fewer adverse events. Here we develop inhibitors targeting a combination of IL-6/sIL-6R and TNF or IL-12/IL-23 signaling, named cs130-TNF^VHH^Fc and cs130-IL-12/23^VHH^Fc. Surface plasmon resonance experiments showed that recombinant cs130-TNF^VHH^Fc and cs130-IL-12/23^VHH^Fc bind with high affinity to IL-6/sIL-6R complexes and human TNFα (hTNFα) or IL-12/IL-23, respectively. Immunoprecipitation experiments have verified the higher ordered complex formation of the inhibitors with IL-6/sIL-6R and IL-12. We demonstrated that cs130-TNF^VHH^Fc and cs130-IL-12/23^VHH^Fc block IL-6/sIL-6R trans-signaling-induced proliferation and STAT3 phosphorylation of Ba/F3-gp130 cells, as well as hTNFα- or IL-23-induced signaling, respectively. In conclusion, cs130-TNF^VHH^Fc and cs130-IL-12/23^VHH^Fc represent a class of dimeric and bispecific chimeric cytokine inhibitors that consist of a soluble cytokine receptor fused to anti-cytokine nanobodies.

Tumor necrosis factor (TNF)α, Interleukin (IL-)6, IL-12, and IL-23 are central immunomodulatory cytokines controlling health and disease (1, 2, 3). Over the years, the general principle of cytokine signaling and receptor complex assemblies have been broadly understood. For IL-6, three receptor assemblies were named classic, trans-, and cluster-signaling. In classic signaling, IL-6 binds to the membrane-bound IL-6R and induces a signal-transducing homodimer of the gp130 receptor chain (4). In trans-signaling, the soluble form of IL-6R (sIL-6R) complexed with IL-6 activates gp130 homodimers on cells lacking IL-6R expression (5, 6). For cluster-signaling, cell–cell contacts by IL-6:IL-6R complexes formed on transmitter cells activate gp130 homodimers on neighboring receiver cells (7, 8).

The sIL-6R-driven IL-6 trans-signaling has been mainly assigned as the pathological mode of IL-6 signaling that contributes to inflammatory, autoimmune diseases and cancer. In many disease models, soluble variants of gp130 (9) were shown to inhibit IL-6 trans-signaling specifically. Treatment with sgp130Fc, the dimeric IgG1-Fc fusion protein consisting of all six extracellular domains of gp130, was shown to be more beneficial compared to global blockade of classic and trans-signaling by neutralizing antibodies including sepsis (10), cerulein-induced acute pancreatitis (11), bone fracture healing (12, 13), and myocardial infarction (14). sgp130Fc was named Olamkicept for clinical development by the WHO in 2016.

At least 0.5% of people in the Western world develop inflammatory bowel disease (IBD) with symptoms like intestinal fibrosis, abscesses, and eventually colitis-related tumors (15). Antibodies blocking TNFα and IL-23 were approved for IBD (16). In contrast, IL-6 antibodies failed in phase II clinical trials for IBD due to non-tolerable side effects (17, 18), including intestinal perforations which were also observed for anti-IL-6R therapy for rheumatoid arthritis (19). Of note, Olamkicept has recently passed two phase II clinical studies for IBD with promising results (20, 21, 22).

We have generated cs130, a size-reduced, highly active, and selective trans-signaling inhibitor (23). cs130 consists only of the first three extracellular cytokine binding domains D1-D3 of sgp130 fused to the a non-neutralizing nanobody VHH6 that binds to IL-6:sIL-6R complexes (23, 24, 25). Based on cs130, c19s130 was the first example of a bispecific trans-signaling inhibitor, which consists of cs130 plus the neutralizing nanobody VHH72 (26) directed against the S-RBD of SARS-CoV2 enabling the inhibition of IL-6 trans-signaling and cellular entry of SARS-CoV2 (27).

Here we refined cs130, resulting in a class of dimeric and bispecific chimeric cytokine inhibitors, which simultaneously block IL-6 trans-signaling and TNFα- or IL-12/23-signaling *via* fusion to nanobodies directed against TNFα (28, 29), and the p40 subunit of IL-12 and IL-23 (30). Such bispecific inhibitors of centrally involved cytokines in IBD might be of therapeutic value for future therapeutic applications, especially once sgp130Fc has been approved for IBD.

## Results

### Development of the bispecific inhibitors cs130-TNF^VHH^Fc and cs130-IL-12/23^VHH^Fc

**Figure 1:**
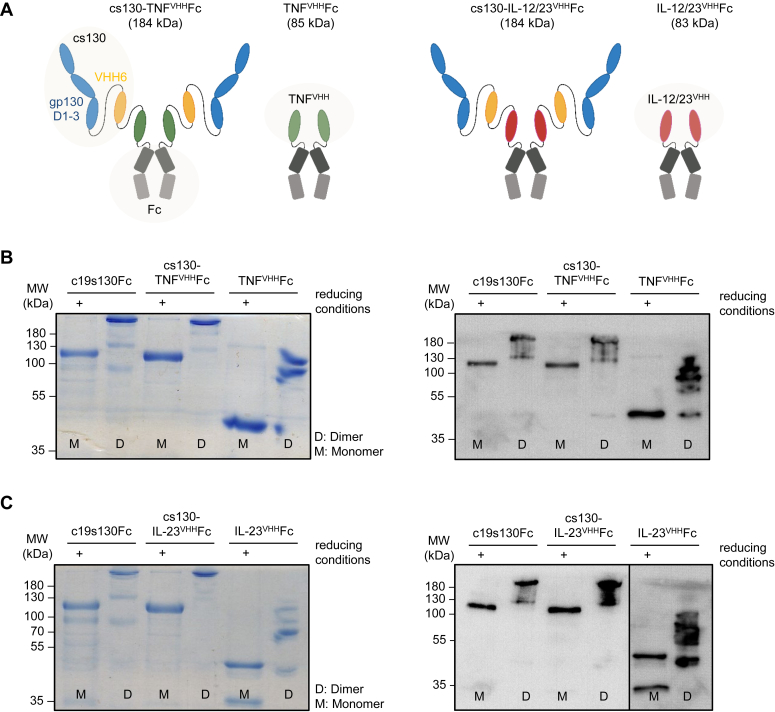
**Generation of cs130-TNF**^**VHH**^**Fc and cs130-IL-12/23**^**VHH**^**Fc.***A*, schematic overview of cs130-TNF^VHH^Fc, TNF^VHH^Fc, cs130-IL-12/23^VHH^Fc, and IL-12/23^VHH^Fc utilized in this study. *B*, SDS-PAGE analysis of purified c19s130Fc, cs130-TNF^VHH^Fc, TNF^VHH^Fc followed by Western blotting (anti-Fc) and Coomassie staining in presence (+) or absence of β-mercaptoethanol. *C*, SDS-PAGE analysis of purified c19s130Fc, cs130-IL-12/23^VHH^Fc, IL-12/23^VHH^Fc followed by Western blotting (anti-Fc) and Coomassie staining in the presence (+) or absence of β-mercaptoethanol.

We designed the two bispecific and dimeric inhibitors cs130-TNF^VHH^Fc, and cs130-IL-12/23^VHH^Fc to simultaneously target IL-6:sIL-6R complexes and human TNFα (hTNFα) (28, 29) or human IL-12/23 (hIL-12/23) *via* the shared cytokine subunit p40 (30). The nanobodies directed against hTNFα and hIL-12/23_p40 (22E11) were described previously (29, 30). We fused the nanobodies for hTNFα or hIL-12/23 to cs130 connected *via* a flexible linker sequence (GGGGS)_2_GGGGTG followed by a C-terminally located IgG1-Fc (Fig. 1*A*). cs130-TNF^VHH^Fc and cs130-IL-12/23^VHH^Fc were expressed and purified *via* Protein A affinity chromatography from supernatants of transiently transfected Expi293F cells. Following affinity purification, cs130-TNF^VHH^Fc and cs130-IL-12/23^VHH^Fc proteins were pure, as demonstrated by SDS-PAGE analysis followed by Coomassie brilliant blue (CBB) staining and Western blotting (Fig. 1, *B* and *C*). The disulfide-mediated dimerization of cs130-TNF^VHH^Fc and cs130-IL-12/23^VHH^Fc was assessed by non-reducing SDS-PAGE stained by CBB and Western blotting (Fig. 1, *B* and *C*). All inhibitory proteins demonstrated a shift to higher molecular weight without a reducing agent, confirming disulfide-mediated dimerization *via* the Fc part. As controls, we also expressed and purified the nanobodies directed against hTNFα and hIL-12/23 with Fc-tag and named the dimeric fusion proteins TNF^VHH^Fc and IL-12/23^VHH^Fc, respectively (Fig. 1, *B* and *C*).

### cs130-TNF^VHH^Fc and cs130-IL-12/23^VHH^Fc efficiently inhibit IL-6 trans-signaling

**Figure 2:**
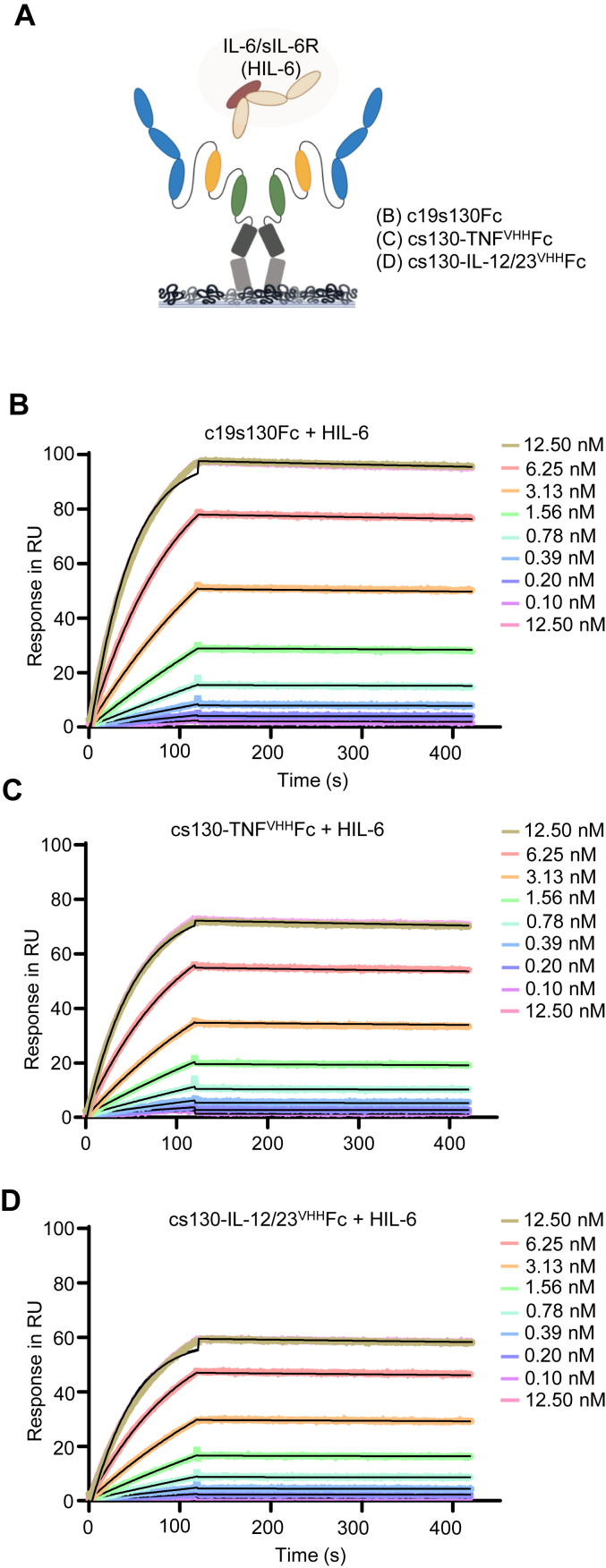
**Binding of cs130-TNF**^**VHH**^**Fc and cs130-IL-12/23**^**VHH**^**Fc to HIL-6.***A*, schematic illustration of surface plasmon resonance experiments. *B*, SPR analysis of c19s130Fc binding to HIL-6. *C*, SPR analysis of cs130-TNF^VHH^Fc binding to HIL-6. *D*, SPR analysis of cs130-IL-12/23^VHH^Fc binding to HIL-6. (*B-D*) c19s130Fc, cs130-TNF^VHH^Fc, and cs130-IL-12/23^VHH^Fc were immobilized on a Protein A chip and increasing concentrations of HIL-6 were injected. Sensorgrams in response units (RU) over time are depicted as colored lines, and global fit data are displayed as black lines.

**Figure 3:**
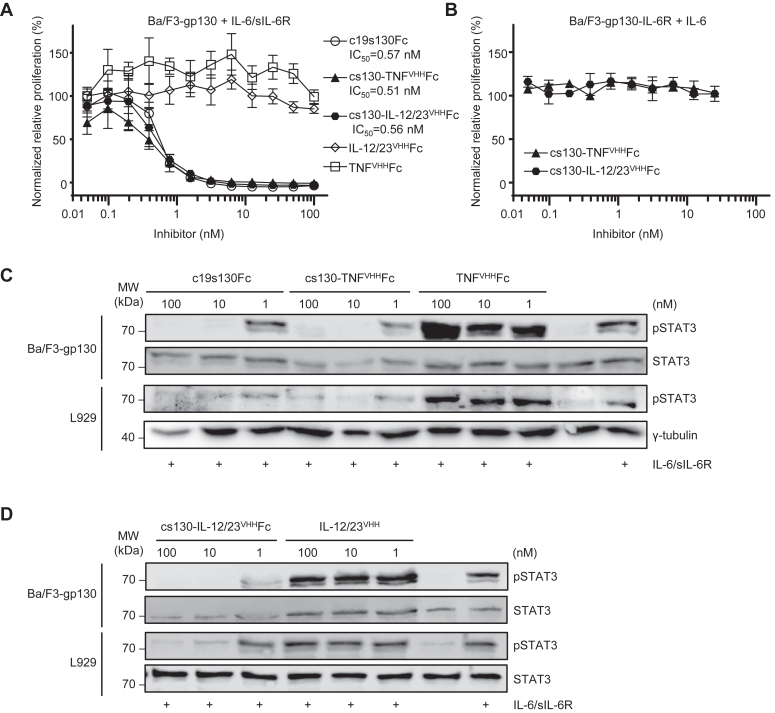
**cs130-TNF**^**VHH**^**Fc, and cs130-IL-12/23**^**VHH**^**Fc block IL-6 trans-signaling.***A*, Ba/F3-gp130 cells were stimulated with 150 ng/ml IL-6 and 300 ng/ml sIL-6R in the presence of increasing concentrations of c19s130Fc, cs130-TNF^VHH^Fc, cs130-IL-12/23^VHH^Fc, IL-12/23^VHH^Fc and TNF^VHH^Fc. 72 h post-stimulation, cellular proliferation was detected using CellTiter-Blue. Data were normalized to HIL-6 control. Assays are representative of three independent experiments. *B*, Ba/F3-gp130-IL-6R cells were stimulated with 10 ng/ml IL-6 in the presence of increasing concentrations of cs130TNF^VHH^Fc and cs130-IL-12/23^VHH^Fc. 72 h post stimulation, cellular proliferation was detected using CellTiter-Blue. Data were normalized to HIL-6 control. Assays are representative of three independent experiments. *C*, Western blot analysis of Ba/F3-gp130 and L929 cells stimulated for 20 min with 150 ng/ml IL-6 and 300 ng/ml sIL-6R in the presence of the indicated concentrations of c19s130Fc, cs130-TNF^VHH^Fc, and TNF^VHH^Fc. Western blots were stained for phosphorylated pSTAT3 and STAT3. Western blots are representative of three independent experiments. *D*, Western blot analysis of Ba/F3-gp130 and L929 stimulated for 20 min with 150 ng/ml IL-6 and 300 ng/ml sIL-6R in the presence of the indicated concentrations of c19s130Fc, cs130-IL-12/23^VHH^Fc, and IL-12/23^VHH^Fc. Western blots were stained for phosphorylated pSTAT3 and STAT3. Western blots are representative of three independent experiments.

The affinities of c19s130Fc, cs130-TNF^VHH^Fc, and cs130-IL-12/23^VHH^Fc to human Hyper-IL-6 (HIL-6) were determined by surface plasmon resonance (SPR). The designer cytokine HIL-6 is a fusion protein composed of IL-6 and sIL-6R connected *via* a flexible peptide linker and is used as a surrogate to induce trans-signaling (31). For all SPR experiments, HIL-6 was used because it forms a stable complex compared to the individual components IL-6 and sIL-6R. c19s130Fc, cs130-TNF^VHH^Fc, and cs130-IL-12/23^VHH^Fc displayed comparably high affinities of 34.2, 48.8, and 28.9 PM for hHIL-6, respectively (Fig. 2, *A*–*D* and Table 1). The kinetic analysis of the interaction revealed the formation of a very stable complex between c19s130Fc, cs130-TNF^VHH^Fc, and cs130-IL-12/23^VHH^Fc with hHIL-6, which was mainly characterized by the low k_off_ rates of 7.5 × 10^−5^ 1/s, 8.6 × 10^−5^ 1/s, and 7 × 10^−5^ 1/s, respectively. Next, we analyzed the inhibitory potential of c19s130Fc, cs130-TNF^VHH^Fc, and cs130-IL-12/23^VHH^Fc towards IL-6 trans-signaling in a cell-based assay. Ba/F3 cells stably transduced with gp130 (Ba/F3-gp130) were stimulated with 150 ng/ml IL-6 and 300 ng/ml sIL-6R, which induced STAT3 phosphorylation-dependent cellular proliferation, a well-established model for IL-6 trans-signaling (25). We observed the concentration-dependent inhibition of IL-6 trans-signaling by c19s130Fc, cs130-TNF^VHH^Fc, and cs130-IL-12/23^VHH^Fc, while no effects were observed for TNF^VHH^Fc and IL-12/23^VHH^Fc (Fig. 3*A*). As a control, cs130-TNF^VHH^Fc, and cs130-IL-12/23^VHH^Fc did not inhibit classical IL-6 signaling induced proliferation of Ba/F3-gp130-IL-6R cells (Fig. 3*B*). Half maximal inhibitory concentration (IC_50_) values of 0.57 nM, 0.51 nM, and 0.56 nM were determined for c19s130Fc, cs130-TNF^VHH^Fc, and cs130-IL-12/23^VHH^Fc, respectively, which were in good agreement with previously published data (23, 27). Ba/F3-gp130 and L939 cells were stimulated with 150 ng/ml IL-6 and 300 ng/ml sIL-6R for 20 min in the presence and absence of the inhibitors c19s130Fc, cs130-TNF^VHH^Fc and TNF^VHH^Fc (Fig. 3*C*) and cs130-IL-12/23^VHH^Fc but not by IL-12/23^VHH^ (Fig. 3*D*) to examine inhibition of signal transduction following IL-6 trans-signaling. As expected, c19s130Fc and cs130-TNF^VHH^Fc but not TNF^VHH^Fc inhibited STAT3 phosphorylation at concentrations above 1 nM (Fig. 3*C*). Furthermore, STAT3 phosphorylation in Ba/F3-gp130 and L939 cells was also inhibited by cs130-IL-12/23^VHH^Fc but not by IL-12/23^VHH^ at concentrations above 1 nM (Fig. 3*D*). Taken together, the fusion of a second nanobody in cs130-TNF^VHH^Fc, and cs130-IL-12/23^VHH^Fc has no negative impact regarding affinity towards IL-6 trans-singaling as shown by biophysical and cell-based assays.

**Table 1:** Surface plasmon resonance analysis of the mono- and bi-specific inhibitors against HIL-6, TNF, and HIL-12

	hHIL-6			hTNFα			hHIL-12	
	c19s130 Fc	cs130-TNF^VHH^Fc	cs130-IL-23^VHH^Fc	cs130-TNF^VHH^Fc	TNF^VHH^ Fc	hsTNFRII-Fc	cs130-IL-12/23^VHH^Fc	IL-12/23^VHH^Fc
KD (pM)	34.18	48.80	28.90	149.9	159.8	92.49	229.5	97.9
k_a_ (1/Ms)	2.190 × 10^6^	1.753 × 10^6^	2.429 × 10^6^	1.115 × 10^6^	1.325 × 10^6^	8.548 × 10^5^	7.098 × 10^5^	1.239 × 10^6^
k_d_ (1/s)	7.483 × 10^−5^	8.554 × 10^−5^	7.019 × 10^−5^	1.671 × 10^−4^	2.117 × 10^−4^	7.906 × 10^−5^	1.629 × 10^−4^	1.214 × 10^−4^

### cs130-TNF^VHH^Fc efficiently inhibits hTNFα-induced apoptosis

**Figure 4:**
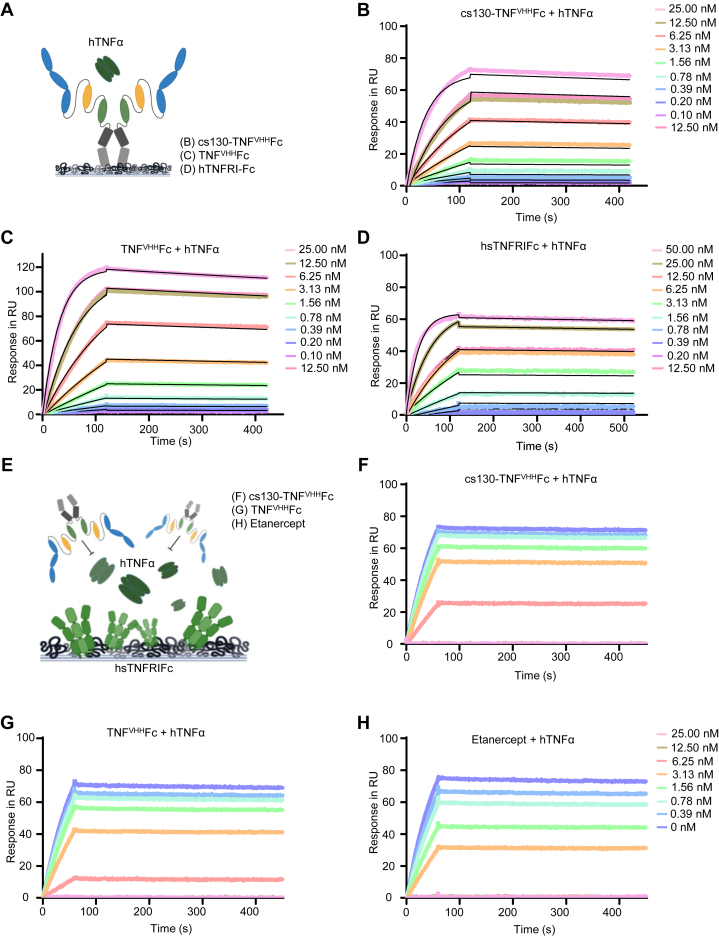
**cs130-TNF**^**VHH**^**Fc blocks binding of TNFα to hsTNFRIFc.***A*, schematic illustration of surface plasmon resonance experiments shown in (*B*–*D*). (*B*) SPR analysis of cs130-TNF^VHH^Fc binding to hTNFα. *C*, SPR analysis of TNF^VHH^Fc binding to hTNFα. *D*, SPR analysis of hsTNFRIFc binding to hTNFα. *B*–*D*, cs130-TNF^VHH^Fc, TNF^VHH^Fc, and hsTNFRIFc were immobilized on a Protein A chip and increasing concentrations of hTNFα were injected. Sensorgrams in response units (RU) over time are depicted as colored lines, global fit data are displayed as black lines. *E*, schematic illustration of surface plasmon resonance experiments shown in (*F*–*H*). SPR analysis of competitive cs130-TNF^VHH^Fc binding against immobilized hsTNFRIFc to hTNFα. hsTNFRIFc was immobilized on a CM-5 chip and hTNFα was injected with increasing concentrations of (*F*) cs130-TNF^VHH^Fc, (*G*) TNF^VHH^Fc, and (*H*) Etanercept. Sensorgrams in response units (RU) over time are depicted as colored lines.

Next, we determined and compared the affinity of cs130-TNF^VHH^Fc, TNF^VHH^Fc, and recombinant human soluble TNFRIFc fusion protein (hsTNFRIFc) (32) to hTNFα by SPR (Fig. 4, *A*–*D* and Table 1). cs130-TNF^VHH^Fc, and TNF^VHH^Fc displayed comparable affinities of 149.9, and 159.8 PM for hTNFα, respectively, demonstrating that fusion to cs130 did not disturb the interaction of the second nanobody with hTNFα. Next, we performed competition experiments with hTNFα bound to hsTNFRIFc with increasing concentrations of the inhibitors by SPR (Fig. 4*E*). hTNFα bound to hsTNFRIFc with 92.49 PM (Fig. 4*D* and Table 1), which agrees with the previously described affinity of 49 PM (33). 12.5 nM of cs130-TNF^VHH^Fc, and TNF^VHH^Fc, whereas 6.25 nM of Etanercept (soluble TNFRIIFc, Enbrel) abrogated the binding of hTNFα to immobilized hsTNFRIFc (Fig. 4, *E*–*H*).

**Figure 5:**
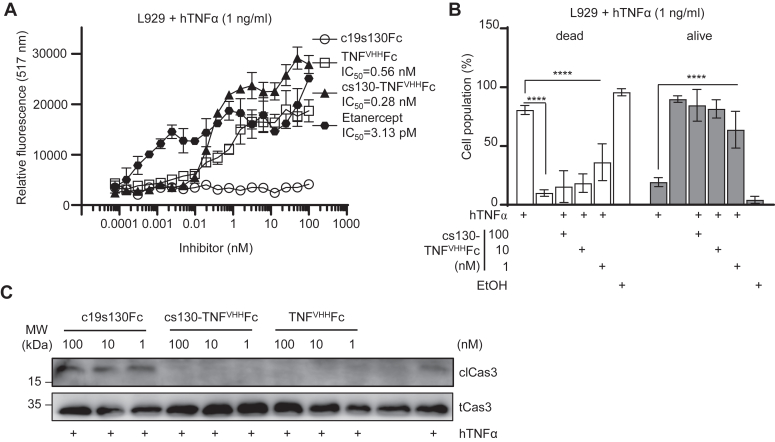
**cs130-TNF**^**VHH**^**Fc blocks hTNFα induced apoptosis of L929 cells.***A*, L929 cells were stimulated 1 ng/ml hTNFα and Actinomycin D in the presence of increasing concentrations of c19s130Fc, cs130-TNF^VHH^Fc, TNF^VHH^Fc, and Etanercept. 24 h post-stimulation, cellular proliferation was detected using the Calcein-assay. Assays are representative of three independent experiments. *B*, L929 cells were incubated for 24 h with 1 ng/ml hTNFα with indicated concentrations of cs130-TNF^VHH^Fc. Controls cells were either untreated or washed with 70% EtOH before the measurement for the EtOH condition. Analysis of cells stained with AnnexinV and 7-AAD by flow cytometry. The mean ±SD of three independent experiments was plotted in the graphs (∗ *p* < 0,05; ∗∗*p* < 0,01; ∗∗∗ *p* < 0,001; ∗∗∗∗ *p* < 0,001). *C*, cleaved Caspase3 in L929 cells treated with 0.1 ng/ml hTNFα in the presence of indicated concentrations of c19s130Fc, cs130-TNF^VHH^Fc, and TNF^VHH^Fc, hTNFα (+) or left untreated (−) for 12 h. Equal amounts of proteins (50 μg/lane) were analyzed *via* specific antibody detecting cleaved Caspase3 and total (uncleaved) Caspase3. Western blotting data show one representative experiment out of three.

Due to the lack of TNF receptors, Ba/F3 cells did not respond to hTNFα. hTNFα can induce cellular apoptosis of L929 *via* activation of TNF receptor I (TNFRI) (34). To demonstrate the inhibition of hTNFα, we chose the L929 cell line which is commonly used for cell death assays. We treated L929 cells with 1 ng/ml hTNFα to determine the inhibitory capacity of cs130-TNF^VHH^Fc, TNF^VHH^Fc, and Etanercept. As depicted in Figure 5*A*, cs130-TNF^VHH^Fc, TNF^VHH^Fc, and Etanercept inhibited hTNFα-induced cell death of L929 in a dose-dependent manner. Notably, the inhibitory profile was comparable with IC_50_s of 0.28 nM, and 0.56 nM for cs130-TNF^VHH^Fc and TNF^VHH^Fc, respectively, whereas Etanercept was significantly better with an IC_50_ of 3.13 pM. Of note, c19s130Fc did not prevent hTNFα-induced cell death of L929 (Fig. 5*A*). Apoptosis of L929 cells was quantified using flow cytometry after 24 h stimulation with 1 ng/ml hTNFα and 1, 10, and 100 nM cs130-TNF^VHH^Fc (Fig. 5*B*). Ethanol treatment served as a positive control for apoptosis, whereas TNFα-non-treated cells served as proliferation control. L929 cells were apoptotic after hTNFα stimulation (81%), whereas inhibition with cs130-TNF^VHH^Fc efficiently prevented apoptosis. About 82 to 84% of cells were still alive when treated with 10 or 100 nM cs130-TNF^VHH^Fc, respectively, but also the lowest concentration of 1 nM cs130-TNF^VHH^Fc showed 64% cell viability (Fig. 5*B*). L929 cells were stimulated with 0.1 ng/ml hTNFα and c19s-130Fc, cs130-TNF^VHH^Fc, and TNF^VHH^Fc for 12 h and cleavage of Caspase 3 (clCas3) from total non-cleaved Caspase 3 (tCas3, pro-Caspase 3) was assessed by Western blotting. Stimulation with hTNFα induced cleavage of pro-Caspase 3, whereas adding cs130-TNF^VHH^Fc or TNF^VHH^Fc largely prevented the occurrence of cleaved Caspase. Cleaved Caspase 3 was almost absent in untreated cells (Fig. 5*C*). Taken together, the fusion and the TNF nanobody in cs130-TNF^VHH^Fc did not interfere with the biological activity towards hTNFα as shown by biophysical and cell-based assays.

### cs130-IL-12/23^VHH^Fc efficiently inhibits IL-12 and IL-23 signaling

**Figure 6:**
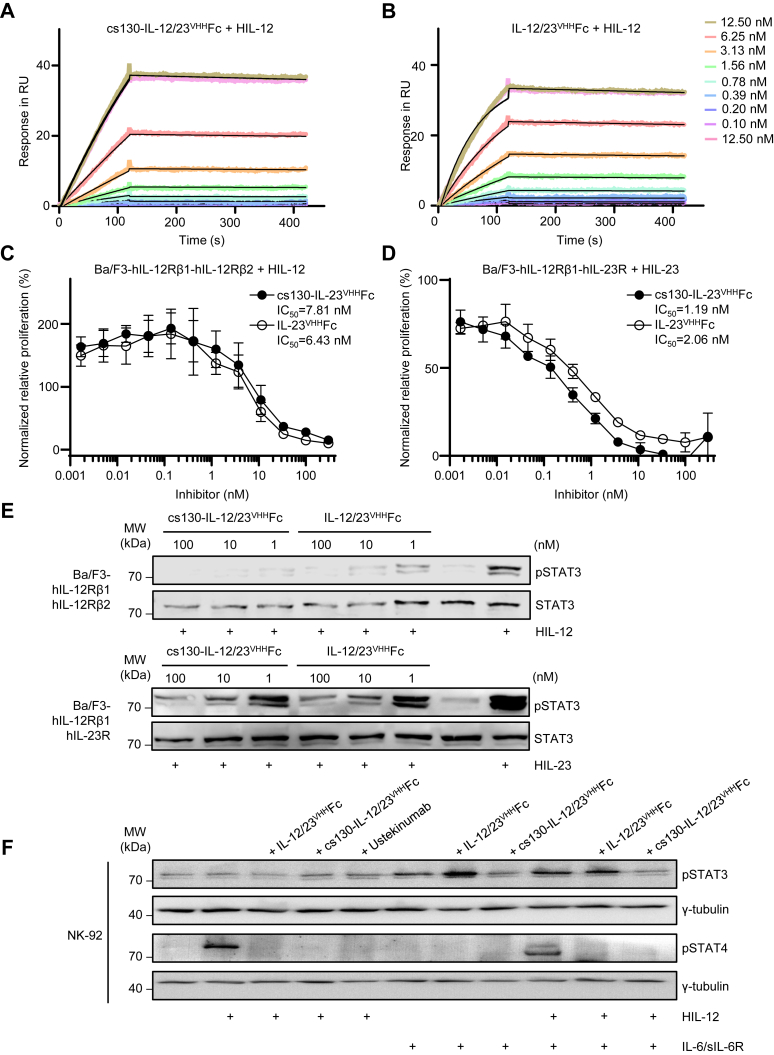
**cs130-IL-12/23**^**VHH**^**Fc inhibits HIL-12 and HIL-23**. *A*, SPR analysis of cs130-IL-12/23^VHH^Fc binding to HIL-12. *B*, SPR analysis of IL-12/23^VHH^Fc binding to HIL-12. cs130-IL-12/23^VHH^Fc, and IL-12/23^VHH^Fc were immobilized on a Protein A chip and increasing concentrations of HIL-12 were injected. Sensorgrams in response units (RU) over time are depicted as colored lines, global fit data are displayed as *black lines*. *C*, Ba/F3- hIL-12Rβ1-2A-hIL-12Rβ2 cells were stimulated with 10 ng/ml HIL-12 in the presence of increasing concentrations of cs130-IL-12/23^VHH^Fc and IL-12/23^VHH^Fc. *D*, Ba/F3-hIL-12Rβ1-2A-hIL-23R cells were stimulated with 0.5 ng/ml HIL-23 in the presence of increasing concentrations of cs130-IL-12/23^VHH^Fc, and IL-12/23^VHH^Fc. 72 h post-stimulation, cellular proliferation was detected using CellTiter-Blue. Data were normalized to HIL-12, and HIL-23, respectively. Assays are representative of three independent experiments. *E*, Western blot analysis of Ba/F3- hIL-12Rβ1-2A-hIL-12Rβ2, and Ba/F3-hIL-12Rβ1-2A-hIL-23R cells stimulated for 30 min with 10 ng/ml HIL-12 or 2 ng/ml HIL-23 in the presence of the indicated concentrations of cs130-IL-12/23^VHH^Fc and IL-12/23^VHH^Fc. Prior to stimulation, HIL-12, HIL-23 and inhibitors were incubated separately for 30 min. Western blots were stained for phosphorylated pSTAT3, STAT3. Western blots are representative of three independent experiments. *F*, Western blot analysis of NK-92 cells stimulated for 30 min with 10 ng/ml HIL-12 or 150 ng/ml IL-6 and 300 ng/ml sIL-6R in the presence of 250 nM cs130-IL-12/23^VHH^Fc and IL-12/23^VHH^Fc. Prior to stimulation, HIL-12, HIL-23 and inhibitors were incubated separately for 60 min. Western blots were stained for phosphorylated pSTAT3, pSTAT4, and γ-Tubulin. Western blots are representative of two independent experiments.

Next, we compared the affinities of cs130-IL-12/23^VHH^Fc and IL-12/23^VHH^Fc to human IL-12/IL-23. Soluble fusion proteins of p40 with p35 (Hyper IL-12, HIL-12), and p40 with p19 (Hyper IL-23, HIL-23) connected *via* a flexible linker served as analytes in SPR. cs130-IL-12/23^VHH^Fc, and IL-12/23^VHH^Fc displayed for p40-related binding in HIL-12 comparable affinities of 229.5 and 97.9 PM, respectively (Fig. 6, *A* and *B* and Table 1). Subsequently, we compared the inhibitory activity of cs130-IL-12/23^VHH^Fc, and IL-12/23^VHH^Fc towards HIL-12, and HIL-23 signaling in cell-based assays. Ba/F3 cells stably transduced with IL-12Rβ1 and IL-12Rβ2 or IL-23R were stimulated with 10 ng/ml HIL-12 or 0.5 ng/ml HIL-23 inducing STAT3 phosphorylation and cellular proliferation. Dose-dependent inhibition of HIL-12-induced cellular proliferation by cs130-IL-12/23^VHH^Fc and IL-12/23^VHH^Fc gave IC_50_ values of 7.81 nM, and 6.42 nM, respectively (Fig. 6*C*). Dose-dependent inhibition of HIL-23 cellular proliferation by cs130-IL-12/23^VHH^Fc, and IL-12/23^VHH^Fc gave the IC_50_ values 1.19 nM, and 2.06 nM, respectively (Fig. 6*D*). Ba/F3-IL-12Rβ1-IL-12Rβ2 cells were incubated with 10 ng/ml HIL-12, and Ba/F3-IL-12Rβ1-IL-23R cells with 2 ng/ml HIL-23 for 20 min in the presence and absence of the inhibitors to determine STAT3 phosphorylation. Both inhibitors, cs130-IL-12/23^VHH^Fc, and IL-12/23^VHH^Fc inhibited STAT3 phosphorylation within the same range at concentrations above 1 nM (Fig. 6*E*). The human natural killer cell line NK-92 was stimulated with HIL-12 and IL-6/sIL-6R. HIL-12-induced STAT4 phosphorylation was inhibited by IL-12/23^VHH^Fc, Ustekimumab and cs130-IL-12/23^VHH^Fc, whereas IL-6/sIL-6R-induced STAT3 phosphorylation was selectively inhibited by cs130-IL-12/23^VHH^Fc but not by IL-12/23^VHH^Fc. Signal transduction after combined stimulation with HIL-12 and IL-6/sIL-6R was, however, only inhibited by cs130-IL-12/23^VHH^Fc (Fig. 6*F*). Taken together, the fusion of cs130-IL-12/23^VHH^Fc did not interfere with the biological inhibitory capacity towards HIL-12, and HIL-23 as shown in biophysical and cell-based assays.

### cs130-IL-12/23^VHH^Fc simultaneously binds to and inhibits IL-6/sIL-6R and IL-23

**Figure 7:**
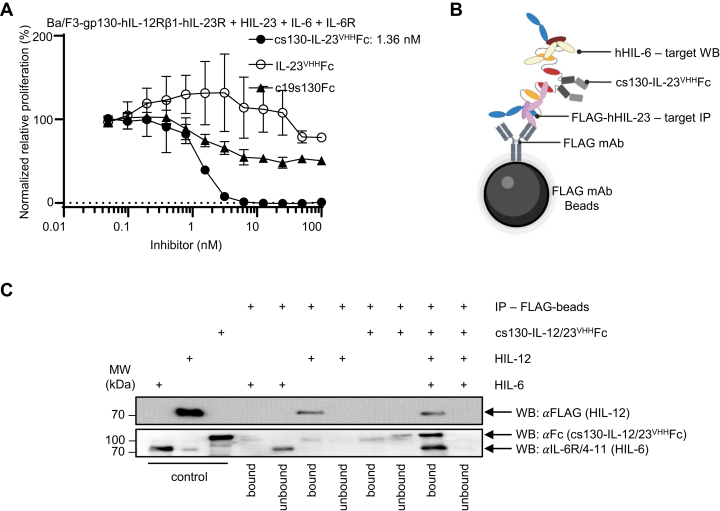
**cs130-IL-12/23**^**VHH**^**Fc inhibits IL-23 and IL-6 trans-signaling simultaneously.***A*, Ba/F3-gp130-IL-12Rβ1/IL-23R cells were stimulated with 0.4 ng/ml IL-23 and 60 ng/ml IL-6 and 100 ng/ml sIL-6R in the presence of increasing concentrations of cs130-IL-12/23^VHH^Fc, c19s130Fc and IL-12/23^VHH^Fc. At 72 h post-stimulation, cellular proliferation was detected using CellTiter-Blue. Data were normalized to HIL-23/HIL-6 control. Assays are representative of three independent experiments. *B*, schematic illustration of the ternary complex formation and the pull-down and detection principle. *C*, pull-down assay of cs130-IL-12/23^VHH^Fc binding to HIL-12 and HIL-6. Recombinant FLAG-tagged HIL-12 was mixed in 1 M ratio with recombinant cs130-IL-12/23^VHH^Fc and recombinant HIL-6. 300 ng of purified protein was loaded for Western blotting and stained with FLAG-, Fc- and sIL-6R-antibodies. Shown is one representative experiment out of two independent experiments.

Ba/F3gp130-IL-12Rβ1/IL-23R cells were cultivated with constant amounts of HIL-6 and HIL-23 plus with increasing concentrations of c19s130Fc, IL-12/23^VHH^Fc and cs130-IL-12/23^VHH^. As shown in Figure 7*A*, cs130-IL-12/23^VHH^ but not c19s130Fc or IL-12/23^VHH^Fc inhibited the proliferation of Ba/F3gp130-IL-12Rβ1/IL-23R with an IC_50_ of 1.36 nM. Furthermore, co-immunoprecipitation (co-IP) verified the formation of a ternary complex with cs130-IL-12/23^VHH^Fc, HIL-6 and HIL-12 (schematic illustration of the co-IP in Fig. 7*B*). In detail, recombinant FLAG-tagged HIL-12 was incubated with recombinant cs130-IL-12/23^VHH^Fc, and recombinant HIL-6 with FLAG-beads. The precipitated bound fraction and the non-precipitated unbound fraction were analyzed by Western blotting (Fig. 7*C*, last two lanes). Western blotting revealed that FLAG-tagged HIL-12 was precipitated with FLAG-beads, which resulted in co-immunoprecipitation of cs130-IL-12/23^VHH^Fc and HIL-6 (Fig. 7*C*, last two lanes). cs130-IL-12/23^VHH^Fc, HIL-6 and HIL-12 were also incubated separately with FLAG-beads as control. Here, only FLAG-tagged HIL-12 was detected in the bound fraction by Western blotting, whereas cs130-IL-12/23^VHH^Fc and HIL-6 remained in the unbound fraction (Fig. 7*C*, lanes 4–9). Lanes 1 to 3 of Figure 7*C* served as loading control using recombinant proteins. Our data demonstrated that cs130-IL-12/23^VHH^Fc simultaneous inhibited IL-6 trans-signaling and IL-23 signaling which is mediated by the formation of a ternary 2xcytokine:cytokine inhibitor complex.

## Discussion

Here, we describe the development of two dimeric and bispecific biomolecules consisting of a basic IL-6 trans-signaling inhibitory module fused to inhibitory nanobodies targeting either hTNFα or IL-12/IL-23 signaling. The basic IL-6 trans-signaling module cs130 is made of the first three extracellular domains D1 to D3 of gp130, which binds IL-6:sIL-6R complexes. Of note, these domains alone facilitate only low-affinity binding (35). High-affinity binding in cs130Fc is achieved by fusion with VHH6, a non-neutralizing IL-6:sIL-6R selective nanobody (23). In contrast to sgp130, cs130 did not inhibit IL-11 trans-signaling (23). Bispecificity toward hTNFα, and IL-12/IL-23 was achieved by the fusion of an additional antagonistic nanobody directed against hTNFα or the shared p40 subunit of IL-12/IL-23 (29, 30), which are located between the IL-6 trans-signaling inhibitor and the Fc part of an IgG1 antibody. The Fc part served as a dimerizer and eased the purification of the two novel cs130Fc variants, cs130-IL-12/23^VHH^Fc and cs130-TNF^VHH^Fc.

Initially, bispecific antibodies (bsAbs), which bind two independent epitopes on the same or different antigens, were developed for therapeutic applications (36). Prominent examples are bsAbs as selective T cell engagers and activators with a binding site for a tumor-associated antigen and CD3 T cell co-receptor. Currently, more than 100 anti-cancer bsAbs are in clinical development, that prevent bacterial and viral infections, interfere with ligand/receptor interaction, mediate intracellular drug delivery, or result in serum half-life extension (37). The bispecific cs130 fusion proteins were highly effective in target binding due to neutralizing IL-6 trans-signaling, TNF-induced apoptosis, and IL-12/IL-23 signaling. We also showed that the cs130-TNF^VHH^-Fc blocks the target cytokines simultaneously without influencing the individual domains, which would reduce the binding affinity.

As mentioned above, sgp130 and variants are potent IL-6 trans-signaling inhibitors largely without affecting classic signaling (9). It seems that sgp130 also blocks inflammatory trans-presentation (cluster signaling), albeit results are still heterogeneous, and blocking might be context-dependent (8, 38). Therapeutic targeting of IL-6 in chronic inflammatory diseases mainly relies on the two IL-6R antibodies tocilizumab and sarilumab, while the siltuximab directed against IL-6 has been approved for Castleman’s disease (39). However, Tocilizumab has failed in phase II clinical trials for IBD due to non-tolerable side effects (17, 18), including intestinal perforations that were also occasionally observed in patients during anti-IL-6R therapy of rheumatoid arthritis (19). A recent publication described the development of bispecific nanobodies targeting IL-6 and TNF with additive efficacy in translational models of rheumatoid arthritis by inhibition of classic and trans-signaling of IL-6 and TNF signaling (40), a strategy that most likely will not work in IBD. The first small open-label phase IIa clinical trial with sgp130Fc (Olamkicept) included 16 patients with IBD (EudraCT No 2016–000205–36). Olamkicept was well tolerated and induced a clinical response in 44% and clinical remission in 19% of the patients (41). The second double-blind placebo-controlled phase IIb clinical study with 91 patients focused on moderate to severe ulcerative colitis (21). Clinical remission was not seen in the placebo group but in 6.7% of the patients receiving the lower dose of Olamkicept (300 mg/injection) and 20.7% receiving the higher dose of Olamkicept (600 mg/injection). Of note, mucosal healing was seen in 3.4% (placebo), 10% (Olamkicept 300 mg/injection), and 34.5% (Olamkicept 600 mg/injection) of the patients (20). Phase III trials are currently in preparation (22). One motivation to generate bispecific cs130Fc variants was based on these recent data suggesting that selective inhibition of trans-signaling might be superior over simultaneous blocking of classic and trans-signaling by IL-6R antibodies in IBD.

We selected nanobodies blocking the activity of hTNFα and IL-12/IL-23 because conventional IgG1 antibodies blocking TNFα and Interleukin (IL-)23 have been approved for the treatment of IBD (16), and therefore these targets are promising candidates to generate small-sized cs130 fusion proteins. Anti-TNFα agents, including Adalimumab, Infliximab, Certolizumab, and Golimumab are now used as biological gold-standard therapy for both colitis ulcerosa and Crohn's disease management (42, 43). The TNF nanobody used in this study is part of Ozoralizumab, a trivalent humanized antibody consisting of two different TNFα nanobodies and a human serum albumin nanobody to increase serum half-life (44). Ozoralizumab was well tolerated and showed efficacy in two recent phase III clinical studies for rheumatoid arthritis (OHZORA and NATSURA trial) (45, 46). The IgG1 antibody Ustekinumab, which targets IL-12/IL-23_p40 subunit, was recently approved for Crohn's disease (47). Like Ustekinumab, the IL-12/IL-23 nanobody used in this study also binds to p40 and prevents interaction of IL-12 and IL-23 with the common IL-12Rβ1 (30).

Taken together, the cs130Fc variants cs130-TNF^VHH^Fc and cs130-IL-12/23^VHH^Fc are the first bispecific cytokine inhibitor fusion protein designs consisting of a soluble cytokine receptor and cytokine-targeting nanobodies.

## Experimental procedures

### Cloning of cs130-TNF^VHH^Fc and cs130-IL-23^VHH^Fc

The cDNAs encoding the single domain antibody TNF-VHH (28, 29) were amplified by PCR using the following forward (5′-ACCGGTGGCGGCGGAGGAAGCAGAGTTCAGCTTCAAGAATCTGGTGGAGG-3′) and reverse (5′-GCGGCCGCAGAAGAAACAGTCACTTG-3′) primer, and 22E11-VHH (30) was amplified using the following forward (5′- ACCGGTGGCGGCGGAGGAAGC GAAGTTCAGCTGGTTGAAAGCGG-3′) and reverse (5′- GCGGCCGCTGAGCTAAC-3′) primer. The cDNAs were subcloned *via* AgeI and NotI in the plasmid pcDNA3.1-Fc (Invitrogen) coding for an N-terminal signal peptide and a myc tag (EQKLISEEDL) and a C-terminal human IgG1-Fc tag. TNF-VHH, and 22E11-VHH were subcloned into pcDNA3.1-c19s130Fc (27) *via* AgeI and NotI to generate cs130-TNF^VHH^Fc, and cs130-IL-12/23^VHH^Fc, respectively.

### Cells and reagents

The generation of Ba/F3-gp130 and of Ba/F3-gp130-IL-6R cells was described (48). Proliferation of Ba/F3-gp130 cells was maintained in the presence of HIL-6 or IL-6/sIL-6R and of Ba/F3-gp130-IL-6R cells by IL-6 (31). Fibrosarcoma cell line L929 was derived from normal subcutaneous areolar and adipose tissue of a 100-day-old male C3H/An mouse. For seeding and subcultivation of L929 cells, cells were first washed with PBS and incubated with trypsin/EDTA solution (Genaxxon bioscience cat. #4261.0110) until cells detached. Cell lines were grown in DMEM high glucose culture medium (GIBCO, Life Technologies) supplemented with 10% fetal bovine serum (GIBCO, Life Technologies), 60 mg/l penicillin and 100 mg/l streptomycin (Genaxxon bioscience GmbH, Ulm, Germany) at 37 °C with 5% CO_2_. Recombinant HIL-6, human IL-6, human sIL-6R and c19s130Fc were produced and purified as described (27). Expi-293F cells (ThermoFisher Scientific) were cultured in 30 ml Expi293F expression medium without antibiotics in shaker flask until they reached a density of 3 to 5 × 10^6^ c/ml in a 37 °C incubator with 8% CO_2_ on an orbital shaker at 125 rpm. NK-92 cells were cultured in RPMI containing 10% fetal bovine serum, 60 mg/l penicillin and 100 mg/l streptomycin at 37 °C 5% CO_2_. Antibodies directed against STAT3 phosphorylated at Tyr705 (clone D3A7), STAT3 (clone 124H6), Caspase-3 (# 9662), and DYKDDDDK Tag (clone D6W5B) were obtained from Cell Signaling Technology. 4 to 11 mAb was produced as described previously (48). Mouse anti-γ-Tubulin (T5326) was obtained from Sigma-Aldrich (Merck KgaA, Darmstadt), rabbit anti-human IgG Fc (#31423) and peroxidase-conjugated secondary Abs (#31432, #31462) were obtained from Pierce (ThermoFisher Scientific).

### Proliferation assays

Ba/F3-gp130 cells were washed three times and 5000 cells were cultured for 3 days in a final volume of 100 μl in the presence of cytokines and inhibitors. The CellTiter-Blue Reagent was used to determine cellular viability by recording the fluorescence (excitation 560 nm, emission 590 nm) using an Infinite M200 PRO plate reader (Tecan, Crailsheim, Germany) immediately after adding 20 μl of reagent per well (time point 0) and up to 120 min thereafter.

### Cytokine stimulation of cells and lysate preparation

10^6^ Ba/F3-gp130 or NK-92 cells/ml were washed and starved in serum-free medium for 5 h. L929 cells were seeded at a density of 5 × 10^5^ cells per 60 mm dish 24 h prior stimulation and also washed five times with PBS before starving in serum-free DMEM for at least 5 h. Prior to stimulation, cytokines and inhibitors were pre-incubated at room temperature for 30 min. Subsequently, cells were stimulated with the indicated cytokines and inhibitor combinations for 20 min, harvested by centrifugation at 4 °C for 1 min at 1500*g*, frozen and lysed. Protein concentration of cell lysates was determined by the BCA Protein Assay (Pierce, Thermo Scientific). Analysis of STAT3 activation was performed by Western blotting of 50 μg of total protein from total cell lysates and subsequent detection steps using the anti-pSTAT3 (Tyr705) (1:1000), anti-STAT3 (1:1000), γ-Tubulin (1:1000), and Caspase 3 (1:1000) antibodies described above.

### Western blotting

Proteins were separated by sodium dodecyl sulfate–polyacrylamide gel electrophoresis (SDS-PAGE) and transferred to nitrocellulose membrane. Membranes were blocked and probed with the indicated primary antibodies. After washing, membranes were incubated with secondary peroxidase-conjugated antibodies (1:2500) or fluorescence-labeled secondary antibodies (1:10,000). The Immobilon Western Reagents (Millipore Corporation) and the ChemoCam Imager (INTAS Science Imaging Instruments GmbH) or the Odyssey Fc Imaging System (LI-CORE Biosciences) were used for signal detection.

### Expression and purification of cs130-TNF^VHH^Fc and cs130-IL12/IL-23^VHH^Fc

Mammalian expression plasmids encoding cs130-TNF^VHH^Fc and cs130-IL-23^VHH^Fc were transfected into Expi-293F cells using ExpiFectamine. Reaching 4.5 to 5.5 × 10^6^ c/ml, the cells were diluted to a final density of 3 × 10^6^ c/ml in 30 ml Expi293F expression medium for transfection. 30 μg of the plasmid expression vectors were used for transfection. Henceforth, the culture was harvested by centrifugation at 450*g* at 4 °C for 5 min, followed by centrifugation of the resulting supernatant at 4000*g* at 4 °C for 20 min. The supernatant of the second centrifugation step was filtered (0.45 μm, Carl Roth cat. #P667.1) and purified by affinity chromatography. Supernatant was loaded on a ProteinA column (1 ml HiTrap MabSelect PrismA; GE Healthcare) at a flow rate of 1 ml/min. The column was then washed with 30 column volumes of PBS. Proteins were eluted at pH 3.2 to 3.5 using a 50 mM citric acid buffer. Fractions containing the protein peak were pooled, and the pH was adjusted to pH 7 with 1 M Tris. Proteins were buffer exchanged to PBS using illustra NAP25 (GE Healthcare Life Sciences) columns. Protein concentration was determined by measuring absorbance at 280 nm, and samples were flash-frozen in liquid nitrogen. 2.5 μg of protein were loaded per lane and separated by SDS-PAGE under reducing (106 mM β-Mercaptoethanol, 95 °C for 10 min) and non-reducing (without β-Mercaptoethanol and cooking) conditions. The gel was stained with Coomassie staining solution (80% ethanol, 20% acetic acid, 4% Coomassie brilliant blue R250) for 1 h and was destained overnight in destaining solution (20% ethanol, 10% acetic acid).

### Surface plasmon resonance

For surface plasmon resonance experiments, the Biacore X100 instrument (GE Healthcare Life Sciences) was used. cs130-TNF^VHH^Fc, cs130-IL12/23^VHH^Fc, TNF^VHH^Fc, and IL12/IL-23^VHH^Fc were captured to a single flow cell of a ProteinA sensorchip to reach 100 response units (RUs) of the analyte at maximal concentration. Three samples containing only running buffer were injected over both ligand and reference flow cell, followed by HIL-6, HIL-12 serially diluted from 50 to 0.1 nM, with a replicate of the 12.5 nM concentration. The analyte was injected at a flow rate of 30 μl/min for 120 s, and the dissociation was measured for 300 s. hsTNFRIFc (R&D Systems, #372-RI-050/CF) was immobilized in 10 mM acetate buffer (pH 5.5) by amine coupling on a CM5 chip (490 RU). After immobilization, hTNFα was injected at a flow rate of 30 μl/min at increasing concentrations (0.2–200 nM). Association was monitored in periods of 120 s, and the dissociation was measured for 400 s. Immobilized hsTNFRIFc was regenerated with 2 M MgCl_2_ to remove bound hTNFα for multiple cycle measurement. 25 nM hTNFα was injected in the presence of increasing concentrations of cs130-TNF^VHH^Fc, TNF^VHH^Fc, and Etanercept (0.2–25 nM). Experiments were carried out at 25 °C in PBS pH 7.4, composed of 137 mM NaCl, 2.7 mM KCl, 12 mM HPO_4_^2−^ und H_2_PO_4_^−^, and 0.05% (v/v) surfactant P20 (GE Healthcare). The resulting data were reference subtracted and fit to a 1:1 binding model using the Biacore X100 Evaluation software V 2.0.1.

### L929-cytotoxicity assays with hTNFα

L929 cells were seeded on a 96-well plate with a density of 30.000 cells/well and cultured for 24 h. Next, the cells were incubated for 30 min at 37 °C with 2.5 μg/ml Actinomycin D (Thermo Fisher Scientific, cat. #15452969). Afterwards, 1 ng/ml hTNFα (Thermo Fisher Scientific, cat. # PHC3011) was added with or without indicated concentrations of cs130-TNF^VHH^Fc, TNF^VHH^Fc, and Etanercept and cultured for 24 h at 37 °C. The cell viability was assessed with Calcein AM (Invitrogen, Thermo Fisher Scientific) following the manufacturer’s instructions on an Infinite M200 PRO plate reader.

### AnnexinV/7-AAD staining

1.25 × 10^5^ L929 cells were used per 6 well and incubated with 1 ng/ml hTNFα with or without indicated concentrations of cs130-TNF^VHH^Fc for 24 h. Cells were washed twice with ice-cold PBS, if indicated with 70% ethanol, and resuspended in 300 μl Annexin V binding buffer (BD Bioscience) with 0.5 μl Annexin V-PE (ImmunoTools) for 15 min in the dark at RT. 1 μl 7-AAD (R&D Systems) was added before analysis *via* flow cytometry recording 20,000 events.

### Immunoprecipitation pulldown assay

Anti-FLAG M2 affinity gel (Sigma Aldrich, FLAG-beads) was washed twice with TBS-Tween (0.05%) at 2700*g* for 2 min. HIL-6, HIL-12, and cs130-IL-12/23^VHH^Fc were adjusted to a concentration of 10 μg/ml, and 15 μg/ml, respectively, in TBS-Tween (0.05%). Proteins were added separately as negative control to 30 μl ANTI-FLAG M2 affinity gel or mixed together in 1 M ratio for pulldown and incubated overnight at 4 °C under gentle agitation. After centrifugation, an unbound fraction was collected, samples were washed twice with TBS-Tween (0,05%), and proteins were eluted with 100 μl 2.5× Laemmli buffer at 95 °C for 10 min 20 μl of supernatant with 300 ng protein was subjected to Western blot analysis.

### Statistical analyses

A representative experiment of n ≥ 3 proliferation assays with comparable results is displayed. IC_50_ values were calculated using a non-linear regression analysis as four parameters variable slope in GraphPad Prism (version 8.0.2 for Windows, GraphPad Software, La Jolla California, United States). The data are presented as means ± SD. For multiple comparisons, two-way ANOVA followed by Bonferroni correction was used (Fig. 5) (GraphPad Prism 8.0.2). Statistical significance was set at the level of graphs *p* ≤ 0.05 (∗ *p* < 0,05; ∗∗*p* < 0,01; ∗∗∗ *p* < 0,001; ∗∗∗∗ *p* < 0,001).

## Data availability

The data that support the findings of this study are available on request from the corresponding author JS.

## Conflict of interest

The authors declare no conflict of interest.
